# Grief reaction, depression, anxiety, and coping of relatives after palliative patients’ death in Thailand

**DOI:** 10.1371/journal.pone.0276583

**Published:** 2022-10-24

**Authors:** Thanita Tantrarungroj, Pornpimon Ocharoen, Veerachai Sachdev

**Affiliations:** 1 Department of Psychiatry, Faculty of Medicine, Ramathibodi Hospital, Mahidol University, Bangkok, Thailand; 2 Department of Family Medicine, Faculty of Medicine, Ramathibodi Hospital, Mahidol University, Bangkok, Thailand; Medical University of Vienna, AUSTRIA

## Abstract

**Introduction:**

Grief is a normal psychological response in relatives after the loss of their loved ones, which has shown to be associated with psychological reactions like depression, anxiety, and significant stress that many relatives have to cope with. In Thailand, there are limited research studies on grief, especially in palliative settings. This study aims to examine grief reaction, depression, anxiety, and coping of relatives after palliative patients’ death.

**Materials and methods:**

A multi-method design was applied. The authors completed the demographic data questionnaire, and the participants finished other measures which included the Hospital Anxiety and Depression Scale (HADS), the Inventory of Complicated Grief (ICG), and the Brief-Coping Orientation to Problems Experienced (Brief-COPE). The qualitative data from the focus group interview was analyzed with thematic analysis.

**Results:**

From the quantitative study, the mean scores of HADS for anxiety and depression subscales were 5.05 and 6.34, respectively, which indicated no anxiety and depressive disorders. The mean score of ICG was 19.51 with highest score on acceptance coping subscale. In contrast, the lowest score was on dealing with the substance subscale. There were significant correlations between anxiety subscale from HADS and ICG (r = 0.73), depression subscale from HADS and ICG(r = 0.85), and anxiety and depression subscale from the HADS (r = 0.79). From the qualitative study, the factors associated with grief reaction could be thoroughly explained according to the perceived character of deceased, perceived character of relatives, relationship characteristics, disease, medical care, and support systems.

**Conclusion:**

The correlations among grief reaction, depression, and anxiety of relatives after palliative patients’ death were high. The grief reaction was associated with many factors, including communication from medical personnel. This finding emphasized the importance of assessing the reactions after loss and associated factors in the relatives after palliative patients’ death. Also, evaluating the ways that the relatives use to cope with their loss, expressing empathy, and supporting the relatives to cope with loss in an adaptive way were recommended.

## Introduction

Grief is a psychological response to the loss of loved ones [1]. It is a natural, universal, and inevitable process of humankind [2]. It is said grief is a myriad of human terrors, the visceral blow that brings rage and outrage simultaneously, the experience of pain and sadness, and the waves of anguish that continue lifelong [3]. A well-known expression of grieving is The Taj Mahal, a monument of love, which was built in 1628 by the grief-stricken emperor Shah Jahan who lost his beloved wife Mumtaz Mahal, who died while giving birth [4].

However, there is variation among people who are grieving. Some individuals experience complicated grief [5], which deviates from normal grief and is associated with impairment in significant areas of health, social, occupational functioning, reduced quality of life, co-occurring with depression and anxiety, and increased morbidity and mortality [6–8].

From past studies, severe grief reaction was associated with many factors; for example, closeness of relationships to the deceased, higher anxiety-related attachment styles, greater dependency behaviors, focus on negative thoughts about the dead, higher emotionality, greater degrees of loneliness, and negative perceptions about the support they will receive if they reach out to others for help [9].

The death of a loved one is considered the most significant stressor for which most bereaved successfully adapt to the circumstances without any clinical intervention. For many, grief may be too much for anyone to go through alone; as seen in the past, many of the most incredible artwork built throughout time is considered an invitation to share grief [10]. Common strategies used for adaptive coping include positive reframing, planning and seeking social support, active coping, emotional and instrumental support, acceptance, religion, and humor. On the other hand, some used maladaptive coping, including venting, denial, substance use, behavioral disengagement, self-distraction, and self-blame [11].

In a palliative setting, grief amongst relatives might be mentioned early to help prepare for the loss. However, there is still little study about suffering in this group. This study aims to examine grief reaction, depression, anxiety, and coping of relatives after palliative patients’ death.

## Method

This study was accomplished using a multi-method design [12, 13]. The authors informed all participants about the study and the consent was obtained from all participants, either by written or verbal method. After that, the authors completed the demographic data questionnaire, and the participants finished other measures. The data was collected and analyzed in descriptive statistics for a quantitative study.

Moreover, to gain a better and deeper understanding of reactions and coping of relatives after palliative patients’ death, the qualitative data was augmented to a quantitative outcome. In addition, the relatives might have a chance to share their experiences and feedback on palliative care from Ramathibodi Hospital, which is essential for improving care for the palliative patients and their families. This study received approval from the Institutional Review Boards COA. MURA2019/1110 on 15 November 2019.

### Participants

Inclusion criteria were

1) Be the direct relatives of patients in palliative service of Department of Family Medicine, Ramathibodi Hospital 2) Being lost from the death of a patient from 1 month to 1 year 3) Were classified as "having abnormal grief" by Ramathibodi Palliative care service 4) Be able to communicate in Thai 5) Be willing to participate in this study.

Exclusion criteria was the authors cannot contact to ask for consent.

Among 229 relatives of patients in palliative service of the Department of Family Medicine, Ramathibodi Hospital, 56 relatives were classified as "having abnormal grief" by Ramathibodi Palliative care service. Of the 56 relatives, 38 met inclusion criteria and were requested to complete the complicated bereavement risk assessment tool, which the Gippsland Regional Palliative Care Consortium developed with permission to use for this study [14].

After the complicated bereavement risk assessment tool was completed for all participants, relatives who received a score of 2 or above were approached to attend an in-depth interview study. A total of 11 participants were willing to participate in the in-depth interview by two authors (TT and VS) at Ramathibodi Hospital or by phone.

### Measures

#### Demographic questionnaire

The demographic questionnaire included age, gender, religion, marital status, educational level, occupation, income, underlying diseases, support system, and loss details.

#### Hospital Anxiety and Depression Scale (HADS)

The Hospital Anxiety and Depression Scale (HADS) is a self-rating questionnaire developed to assess depression and anxiety in patients for both hospital and community settings. It consists of 14 items equally divided into two subscales: anxiety (HADS-A) and Depression (HADS-D). Responses are rated on a 4-point Likert scale and range from 0 to 3 [15]. Recommended cut-off scores are 8–10 for doubtful cases and 11 or above for definite points [16].

The Thai HADS had good reliability and validity for both anxiety and depression subscales. At the cut-off point of > 11, the best cut-off point, the sensitivity of anxiety and Depression subscales of Thai HADS were 100% and 85.71%, respectively. In comparison, the specificity was 86.0% for anxiety and 91.3% for depression. Both sub-scales also showed good internal consistencies with Cronbach’s alpha coefficient of 0.8551 for the anxiety subscale and 0.8259 for the depression subscale [17].

#### Inventory of Complicated Grief (ICG)

The Inventory of Complicated Grief was developed by Prigerson et al [18]. Exploratory factor analyses indicated that the ICG measured a single underlying construct of complicated grief. High internal consistency and test-retest reliability were evidence of the ICG’s reliability. The ICG total score’s association with the severity of depressive symptoms and a general measure of grief suggested a valid yet distinct assessment of emotional distress. Respondents who score over 25 are considered high risk for requiring clinical care (APA) and are significantly more impaired in social, general, mental, and physical health functioning and bodily pain [19].

The ICG was permitted to translate and adjust to Thai culture by Orasa Yaiyong and Peeraphon Lueboonthavatchai. It has good validity and reliability with Cronbach’s alpha coefficient of 0.97 [20].

#### Brief-Coping Orientation to Problems Experienced (Brief-COPE)

The Brief-COPE is a self-report questionnaire developed to assess a broad range of coping responses. It has 14 subscales: acceptance, emotional support, humor, positive reframing, religion, active coping, instrumental support, planning, behavioral disengagement, denial, self-distraction, self-blaming, substance use, and venting [11].

The Brief COPE inventory-Thai version has acceptable concurrent validity (Pearson correlation coefficient with Proactive Coping Inventory = 0.25–0.45) with high internal consistency (Cronbach’s alpha coefficient = 0.70) [21].

### Data analysis

Descriptive statistics were performed using IBM SPSS version 26. Demographic data, scores of the HADS, ICG, Brief-COPE were analyzed and presented in Mean±SD and correlation among variables. The qualitative data consisted of an interview guide administered to focus groups. The qualitative data was analyzed which consisted of thematic analysis proceeding through several steps to generate the themes. The van Manen approach was used in the thematic analysis to perform a phenomenological qualitative study. The broad aim of such research is to interpret the meaning of phenomena and to understand the lived structures of meanings. There are four steps in the process. 1) Preparing the interview transcript and checking the accuracy of the transcripts by repeat listening to the recording and reading the transcripts until an interview transcription is accurate. 2) Isolating thematic statements using holistic, selective, and detailed approaches. 3) Composing linguistic transformations via writing, rewriting, and generating illustrative examples connected with the phenomenon of interest. 4) Development of descriptions explaining themes while maintaining the essence of the phenomenon of interest [22].

## Results

### Demographic data

Details for demographic data of participants were presented in Table 1.

**Table 1:** Demographic data.

Characteristics		N(%)
Gender	Male	9(22.5)
	Female	31(71.5)
Religious	Buddhism	39(100)
Hometown	Bangkok Metropolitan Region	30(75)
	Central region	4(10)
	Eastern region	3(7.5)
	Northern region	2(5)
	Northeastern region	1(2.5)
Marital status	Single	15(37.5)
	Married	19(47.5)
	Widowed	4(10)
	Divorced	2(5)
Education level	Secondary education and lower	5(12.8)
	Bachelor’s degree	31(79.5)
	Higher than bachelor’s degree	3(7.7)
Occupation	Unemployed	6(14.6)
	Employed	33(85.4)
Income per month	Less than 20000 THB	9(25)
	20000 to 40000 THB	14(38.9)
	More than 40001 THB	13(35.1)
Support system	Yes	35(87.5)
	No	5(12.5)
Relationship with the deceased	Spouse	8(20)
	Parents	8(20)
	Child	18(45)
	Sibling	3(7.5)
	Others	3(7.5)
Deceased’s cause of death	Malignancy	31(77.5)
	Others	9(22.5)
Deceased’s length of stay before death	27.15 ± 23.13 (0–87) days	
Realized about cancer from	Routine checkup	5(12.8)
	Abnormal symptoms	34(87.2)
The suddenness of the loss	Sudden	24(60)
	Gradual	16(40)
The agreement in the relationship	Agreement	38(95)
	Disagreement	2(5)
Closeness in the relationship	Deeply Connected	36(90)
	Moderately connected	3(7.5)
	slightly connected	1(2.5)

### Quantitative examination

From HADS, the mean scores of HADS for both anxiety and depressive subscales (5.05 and 6.34, respectively) indicated no anxiety disorders and depressive disorders in participants. By ICG scoring, the mean score of ICG in participants was 19.51.

The highest mean score of Brief COPE was on item 24 (mean 3.46), followed by item 20 (mean 3.46), in which both things are within acceptance coping subscales. In addition, the two lowest scores were on item 11(mean 1.15) and item 4(mean 1.17) in dealing with the substance subscale. Data are shown in Table 2.

**Table 2:** Total scores of Inventory of Complicated Grief (ICG), Hospital anxiety and depression scale (HADS), and the Coping Orientation to Problems Experienced Inventory (Brief COPE).

Measures		Mean ± SD
Inventory of Complicated Grief (ICG)		19.51 ± 18.68
Hospital anxiety and depression scale (HADS)	Depression subscale	6.34 ± 6.48
	Anxiety subscale	5.05± 4.82
The Coping Orientation to Problems Experienced Inventory (Brief COPE)	Active coping subscale	5.48 ± 1.99
	Planning subscale	5.10 ± 2.21
	Positive reframing subscale	5.83 ± 1.87
	Acceptance subscale	6.90 ± 1.55
	Humor subscale	3.54 ± 1.81
	Religion subscale	5.56 ± 1.92
	Emotional support subscale	5.63 ± 1.77
	Instrumental support subscale	5.73 ± 1.95
	Self-distraction subscale	5.71 ± 2.10
	Denial subscale	3.17 ± 1.83
	Venting subscale	4.24 ± 1.50
	Substance use subscale	2.32 ± 0.85
	Behavioral disengagement subscale	3.27 ± 1.70
	Self-blaming subscale	3.88 ± 2.04

In terms of the correlation between HADS and ICG, the results showed a significant correlation between anxiety subscale from HADS and ICG (r = 0.73, p = 0.00), depression subscale from HADS and ICG(r = 0.85, p = 0.00), also, between anxiety and Depression subscale from the HADS (r = 0.79, p = 0.00).

Correlation between ICG, HADS, and Brief-COPE were shown in Table 3.

**Table 3:** Correlation between ICG, HADS, and Brief-COPE.

	ICG	HADS-D	HADS-A	BC1	BC2	BC3	BC4	BC5	BC6	BC7	BC8	BC9	BC10	BC11	BC12	BC13	BC14
ICG	1	0.85* 0.00	0.73* 0.00	-.28 0.08	-0.8 0.621	-0.35*0.03	-0.65* 0.00	-0.43*0.01	0.05 0.78	-0.31* 0.05	-0.19 0.25	-0.42* 0.01	0.76* 0.00	0.49* 0.00	0.36* 0.02	0.44* 0.00	0.75* 0.00
HADS-D	0.73* 0.00	1	0.79* 0.00	-0.31 0.06	-0.62 0.70	-0.06 0.70	-0.64* 0.00	-0.51* 0.00	0.08 0.63	-0.32* 0.04	-0.13 0.41	-0.51* 0.00	0.70* 0.00	0.34* 0.03	0.20 0.21	0.28 0.07	0.69* 0.00
HADS-A	0.73* 0.00	0.79* 0.00	1	-0.19 0.24	-0.06 0.69	-0.06 0.69	-0.64* 0.00	-0.45* 0.03	-0.03 0.84	-0.44* 0.00	-0.32* 0.04	-0.48* 0.00	0.45* 0.00	0.33* 0.04	0.33* 0.04	0.17 0.28	0.55* 0.00

ICG = The Inventory of Complicated Grief total score, HADS-D = Hospital Anxiety, and Depression Scale- Depression subscale, HADS-A = Hospital Anxiety, and Depression Scale- Anxiety subscale

BC1 = Brief-COPE active coping subscale, BC2 = Brief-COPE planning subscale, BC3 = Brief-COPE positive reframing subscale, BC4 = Brief-COPE acceptance subscale, BC5 = Brief-COPE humor subscale, BC6 = Brief-COPE religion subscale, BC 7 = Brief-COPE emotional support subscale, BC 8 = Brief-COPE instrumental support subscale, BC 9 = Brief-COPE self-distraction subscale, BC 10 = Brief-COPE denial subscale, BC 11 = Brief-COPE venting subscale, BC 12 = Brief-COPE substance use subscale, BC 13 = Brief-COPE behavioral disengagement subscale, BC 14 = Brief-COPE self-blaming subscale

✱ = p < 0.05

### Qualitative examination

Factors associated with grief reaction could be thoroughly explained according to perceived character of the deceased, perceived character of relatives, relationship characteristics, disease, medical care, and support systems.

### Factor related with the deceased

#### Characters of the deceased

Most relatives said that the deceased was friendly and had a positive impact on their life. Many were known as people-pleasers, while some would always do things for others, making them well-loved by everyone.

For example, participant V2 said, "… ..My husband is a very good man, and he always helps other people, and everyone loves him. He loved me and constantly took care of me throughout our 40 years period until his death…"

Another participant, V3, said, "…I feel like I cannot accept the loss…because she is so nice… really nice. She always smiles and has mellifluous words…she never argues with others. Also, she always pays respect to her parents and mine… .and she does it every day. She told me to give money to my parents every month, whether more or less. She practices dharma, prays every day, and goes to a meditation retreat every month.”

### Factor related with the relatives

#### Characters of the relatives

Being dependent on close ones, whether physical, mental, or financial, was related to a worse grief reaction when experiencing the loss. One participant, T6, said, "… .We lived together for 30 years…he never left me alone, he takes care of me for everything…illness, food, clothes…he takes me outside every day to make me feel fresh. I’m handicapped with knee ligament fixation, so if I do not wear knee support, I cannot walk straight…And he always takes care of me on this matter.”

Another participant, T1, said her mom helped her with a loan payment for a car. “During my financial difficulties, she supported me financially.”

#### The relative’s experience about loss

History of loss without a chance to say goodbye may worsen the grief reaction. For example, participant T1 said she lost her father from cancer "… ..When I went to the hospital, my dad was already on a ventilator…he could not speak, and he did not have a chance to admonish at last…and he died on that day… .he tried to talk, but everyone forbade him……. It’s still an unresolved issue for me.”

On the other hand, participant T2 said the experience of losing his parents helped him cope with the current loss of his wife…. “I abruptly lost my dad, but my mum was in a hospital for many days, with oxygen support before she left me. I knew that one day I would lose my parents, and I have had that experience. When it was my wife’s turn, I felt that I used to cope with losses and I would get through this…”

#### Opportunity for preparing for the loss

Many survivors said about not having a chance to talk to their loved ones about forthcoming death because they were concerned about the patients. Participant T3, said "….I did not prepare to lose my daughter…even the doctor told me to do so. What would my girl think about this? I cared about her feelings. I would not talk about this approaching death to her and prepare nothing for the coming loss. I hoped she would get better."

By contrast, some participants said they did not get comprehensive information about their loved ones’ illnesses. It is the reason why they did not have conversations about preparing with the patients. For example, participant T5 who lost her daughter, said, "…I did not know about her illness. If I knew in the first place…She felt sick for two days before being admitted to the hospital. Her brother was with her, but no one told me. Her boyfriend called me to ask whether I knew this. I thought she knew her condition… quite well…she told others but not me…she did not want me to worry with her illness."

#### Unresolved issues

Some participants said they were still disturbed by the thought that they could not fulfill the deceased’s expectations. One participant who lost her mother, T1, said, "…My mom repeatedly told me about getting married since I broke up with my ex and was in a relationship with a new guy. She asked me often whether my boyfriend would have a serious relationship or not, would he get married to me? She was so serious about the wedding. I felt that I did not do this when my mom was still alive…just one thing that she expected from me…Why could I not do this for her?”

### Factor related with the characteristics of a relationship

#### Relationship with the deceased

Many participants said they had a very close relationship with the deceased. Participant T3, said "…I looked after her (his daughter) and did everything for her. I took her to school and picked her up every day until she was in grade 7….I slept near her school while she was studying in Bangkok. I have to keep an eye on her.”

On the other hand, one participant, T8, talked about the history of separation from a significant person, her mother, for a very long time. She said…. "I did not live with my mom. I wondered why I had to live with my aunt, who had to manage everything for me…Then, in 2001, I had a chance to stay with my mom, and hug her. It made me so happy, and it made me want to be with her to make up for the period of separation."

### Factor related with the illness and medical care

#### Perceived progression of the illness

Although most deceased had a chronic illness, mainly cancer, the relatives might feel that the death happened suddenly. Participant T7said, "…I think it was not slow, it’s fast… I’m still confused why it was so fast…"

#### Perceived about not receiving the utmost care

Some participants thought that their beloved ones might have lived longer if they got better medical treatment. For example, participant T5 said, "…From the bottom of my heart, I wonder if the doctor made a mistake…cause my girl was still well…It was late in receiving treatment, and many medical students came in for interviewing…If she admitted to the previous hospital, she might be still alive”

In the same way, participant T7 said, "…Nowadays I still in doubt about the received treatment, and I wonder if the more experienced doctor might have additional treatment for my daughter…It has been bothering me. Also, doc did not visit my daughter after he allowed her to go home, but I asked for a more extended stay…and the nurse did not frequently visit my daughter.”

#### Miscommunication from medical staffs

One participant, T3, said, "… ..the treatment plans from medical staff were inconsistent…. one said about chemotherapy, another said about pleural tapping, and another said about just taking oral medicine the patient would get better."

Some participants felt they were repelled from the hospital. Participant T3 said, "…this doctor wanted to kick out my daughter from the hospital…he said that to me three times…he said "go to your universal health coverage’s hospital "…and I thought my daughter could claim for medical treatment expense… and my girl was in critical condition and could not look after herself…I don’t understand…when he sped up the admission time…he might give drugs that made her death come faster…no encouragement at all…he treated me like villagers…dunces…He asked my son whether we had a plan of the place of her death.”

In addition, participant V2 commented, "… .I know, Sleeping or lying down might be impolite. However, relatives surely want to be with the patients in critical moments. Secondly, some of them do not have money to rent a place to stay. Sometimes I saw the relatives were asked to leave the hospital… and I felt terrible about this situation.”

#### Being forced to decide for the patients who were in critical condition

In addition, participant V2 criticized feeling utterly repressed from being forced in decision making in a patient’s emergency condition. She said, "… ..and then my husband was moved to the resuscitation room. The doctor asked me whether I had decided to sign the "Do not resuscitate" request while I was hoping that he was just tired…I was forced to answer immediately…he said, "you had to sign for approval or refusal for resuscitation…It was cruel…really cruel…even I walked out the room, he chased me and relentlessly asked for the answer….he said "You have to know that he is dying and so on….I understand thoroughly….but I need to get a grip for making the decision…the doctor might get used to this…but the ordinary people who experienced this situation…it was not normal at all…and the doctor squeezed me to answer and told me to ask the patient…he was unconscious, right? But you had to discuss the pros and cons of intubation. It was the pressure to me in making a decision. I’m not ready…deeply not. I felt terrible…then I talked to another doc, and he said no, not this time. First, we will use the mask first, if he were OK, we would decide on intubation later, and then I felt OK.

However, it’s not OK for me at that time. That doc ordered me to sign the form immediately. Don’t you think it is too cruel?..It was a soul-sucking decision….If there is anything I would like the hospital to empathy…it would be the difficulty for patients and relatives to make the decisions…."

#### Perspective about palliative care

Participant T4 commented that "… . It’s like the repeated word was briefed to me whenever the team came in….. ’It was the symptoms of disease….the disease was this and that…I was concerned with my dad’s eating difficulty, but I was briefed that it was a symptom…it was blocked by the word "end of life"…We can do nothing, and more procedures might get him hurt and suffer. I could not accept it at that moment, and I neither studied nor tried to understand palliative care…After my father’s death and I searched for information about that, I began to understand and did not regret the hospital. Every personnel did their job…The information made me feel better.”

In the same way, participant T7 said, "…I found the information about palliative care and this made me feel better…it told me that my decision was not wrong.”

**Effects from COVID-19 pandemic.** Participant V3 said, "… .I felt the distance…I could not have a chance to stay close to her or give her encouragement. I accused COVID-19 of making us apart, and my girl became upset.”

### Factor related with the support system and spiritual anchor

#### Support from relatives and surrounding people

Participant T5 said, "…I still have another child, my son. If not, I don’t want to live, really don’t want to live, doctor. Also, participant T1 commented, "… .I received much support from my relatives. They helped me to find many activities to do and spend time together, like seeing movies, playing with my grandchildren, distracting me from the lost, talking only good things…."

On the other hand, participant T6 commented, "…my late husband’s siblings wanted to take many things from me. They didn’t understand that his brother had his own family…..They want my house, my money, my properties, and so on. Moreover, participant V2 said, "…I didn’t want to hear "cheer up!" or "It’s a normal part of life"…something like this from other people…..I felt angry…cause I surely knew what would happen at the end…Even if I gave up, I must face it, right? They should say nothing. But touching, hugging, or touching hands and just saying, "Are you OK?" Those physical communications helped me.”

In addition, the feeling of having inadequate support made the grief worse. For example, participant T6 said, "… ..I have no one, and I don’t know where to get help….they just said It’s boring…they didn’t want to hear…I didn’t tell many people and didn’t want them to be disturbed.”

#### Spiritual anchor

Participant T3 said, "…I offered sacred marker round stone ("Luuk Nimit") and boundary stone ("Sima") and wrote my daughter’s name. Moreover, after the funeral, I copied 1000 prayer books for giving to others. In the morning, I offer food to monks and pour ceremonial water for dedicating merit to her.”

Also, participant V2 said, "Buddhism helped me a lot. I don’t mean that going to the temple only in suffering time. We should practice our mind since we are OK….to cope with the problem….you have to be mindful and let it go… I’m sensitive…so others might take one month or one year to forget…I believe I need triple…and I also offer food to monks every morning and dedicate merit to him.”

## Discussion

The objectives of this study were to examine psychological reactions and coping of relatives after palliative patients’ death using multi-method study design. From the result of the quantitative section, the total score of ICG indicated that the grief reaction was not severe [18].

It was similar to the scores of depression and anxiety subscales of HADS, which were regarded as being in the normal range [23]. Also, the total score of ICG was correlated with the depression and anxiety subscales, which was consistent with a previous study [24].

According to the coping styles used by the relatives, the top three-highest rated coping styles were Acceptance, Positive reframing, and Instrumental support subscales, respectively, which indicated the adaptive coping styles. These might explain the low scores of the total ICG and HADS scores.

Moreover, the total of ICG was negatively and significantly associated with some adaptive coping, such as positive reframing, acceptance, humor, and using emotional support. In contrast, it significantly correlated with some maladaptive coping, such as denial, substance use, behavioral disengagement, and self -blame.

Deeper understanding gained from qualitative examination about factors related to worsened grief reaction, which included factors related with character of the deceased, character of the relatives, the characteristics of a relationship, the disease and medical care, and support systems.

From this study, the characters or personalities of the deceased and the relatives affected the grief reactions. These were consistent with a previous study in the past [25], which stated that habitual styles of perception, thought, coping, and defense determine how a person experiences and handles all life situations, including the stress of bereavement. Also, people who are characteristically more flexible and able to use more mature coping strategies will deal with bereavement more effectively than others.

Characteristics of a relationship is another important factor related to grief reaction. From this study, the two most prominent kinds of relationship that affected the grief reaction were that the relatives were being dependent on the deceased and had a very close relationship with the patient, which was seen in the previous study [25].

Communication between medical personnel and the relatives is very important and has a tremendous impact on the relatives [26]. Unclear and inadequate explanations about the progression of the disease, treatment options, and late introduction to palliative care might lead to the perception about not receiving the utmost care, especially in the time that the relatives had to make an important decision, like Do-Not-Resuscitate (DNR) consent.

It was shown in this study that not expressing empathic responses from the medical team and not giving adequate time for decisions making to the relatives made a significant negative impact on the relative’s grief reaction.

Receiving support from others, both from their relatives and surrounding people has shown to lessen the grief reaction. Also, having spiritual anchors helped alleviate the relatives’ grief reaction as well [27]. In this study, performing good deeds helped the relatives in the way that they can do some good things for the departed.

Lastly, during the COVID-19 pandemic was a tragic moment for the relatives. Many did not have a chance to look after their loved ones during the last days of life, whilst some did not get an opportunity to say their last goodbye. Applying the preventive measure to lessen the risk of infection while honoring loved ones’ wishes and cultural traditions before separation should be considered [28, 29].

To sum up, the grief reaction from the relatives could be lessened by giving adequate information about palliative care from the medical team and by giving enough time for relatives to accept palliative care along with making life-threatening decisions. In addition, properly and gradually introducing palliative care treatment, including advance care plan while the palliative patients still alive and concurrently receiving curative treatment, might help in improving care delivered to the patients and their relatives. Lastly, this result suggested that the palliative care team should explore the relatives’ reactions after loss and associated factors, the ways that they use to cope with their loss, express empathy, and support the relatives to cope with loss in an adaptive manner.

### Limitation

There are a few limitations to this study. First, the coming of the Covid-19 pandemic during the middle of this study made it more challenging for the participants to come to the hospital for the interview. The authors had to adapt the interview process where many participants were interviewed by phone, which was an obstacle to good communication. Secondly, the relatives’ factors such as financial problems and transportation from distant areas, discomfort to talk about the loss from physical and psychological aspects, and dislike about facing the triggers in the hospital. Also, inaccurate data in the medical record interrupted the communication to the relatives. In addition, low education might be a problem for the relatives to explain their experience about loss accurately.

### Suggestion

There should be a widely acceptable policy in providing palliative care services for all hospitals, and health providers should encourage patients to discuss their own living wills including advance care planning, particularly for palliative cases. Moreover, relatives should be given an opportunity to take compassionate care leave while caring for their loved ones during the final weeks of life.

Communication is very crucial for relatives. The health care team should express empathic responses, not only giving information about the disease. Moreover, the team should be careful in selecting words in the conversation and aware of adverse reactions from the relatives.

In regards to Informed consent, health providers should be trained in providing necessary information, where relatives should be given enough time to make decisions about life sustaining measures, along with an opportunity to ask questions without feeling pressured into signing consent forms and providing a channel for providing feedback from the relatives might help lessen their negative emotions.

Grief screening should be provided for all deceased’s relatives, not only for assessed high-risk groups. Also, a high alert system for referring suspected complicated grief to psychiatric service would be beneficial.

Lastly, providing a resource channel for further care, through a hotline, whatsapp, line contact ID or other valuable sources of information, might help relatives cope with grief by knowing where they may seek support if needed.

### Further study

It might be helpful to ask for more details about relationships in the family which will be accommodated as an early warning system for upcoming severe grief reactions. Moreover, it might be worth considering providing palliative care in the intensive care unit (ICU), which has a high mortality rate. It encompasses communication with relatives which might help alleviate the grief reaction [30, 31].
